# The Dietary Lipid Requirement for Ovarian Maturation and Health in Female Giant River Prawn, *Macrobrachium rosenbergii* Broodstock

**DOI:** 10.1155/2024/7462841

**Published:** 2024-11-05

**Authors:** Jiaxin Song, Yonghui Jian, Yuliang Xie, Jinghao Liang, Chaowei Shao, Xifang Pan, Zhiyuan Chen, Qiuyu Gao, Youqin Kong, Qiyou Xu, Zhili Ding

**Affiliations:** Zhejiang Provincial Key Laboratory of Aquatic Resources Conservation and Development, College of Life Science, Huzhou University, Huzhou 313000, Zhejiang, China

**Keywords:** broodstock, histology, lipid level, *Macrobrachium rosenbergii*, ovarian development

## Abstract

The dietary lipid level is closely associated with ovarian maturation of broodstock. However, optimal lipid requirements during broodstock gonad development for aquatic animals remain limited. In order to assess the impact of dietary lipid levels (6%, 8%, 10%, 12%, and 14% lipid, denoted as L6%, L8%, L10%, L12%, and L14%) on the ovarian maturation, antioxidant status, and messenger RNA (mRNA) expression of genes involved in the lipid metabolism of *Macrobrachium rosenbergii* broodstock (initial weight 10.53 ± 1.97 g), this study carried out an 8-week feeding experiment. The findings showed that while there was no significant difference in the survival rate across the groups (*p* > 0.05), the weight gain observed in prawns fed the 8% lipid-level diet was significantly higher than those fed other diets (*p* < 0.05). The hepatosomatic index and the gonadosomatic index showed a significant increase with the rise in dietary lipid level (*p* < 0.05). More ovaries from *M. rosenbergii* broodstock reached stages Ⅲ and Ⅳ after being supplemented with dietary lipid levels between 8% and 14%. Serum glucose content did not show any significant difference among all groups (*p* > 0.05), but serum triglyceride and total cholesterol content increased followed by a decreasing trend with increasing levels of dietary lipids, both peaking in the prawns fed a 10% lipid-level diet. Furthermore, the progesterone (PROG) and 17*β*-estradiol (E_2_) content of prawns fed the 10% and 12% lipid-level diets were significantly higher compared to other groups (*p* < 0.05). Based on serum E_2_ and PROG content, the optimal lipid level needed for maximal ovarian maturation was determined to be 11.79% and 10.88%, respectively. Moreover, there were more endogenous vitellogenic oocytes in prawns fed 8% and 10% lipid-level diets, with a more compact arrangement compared to the less tightly arranged structure of the ovarian tissue in prawns fed other diets. With the increase in dietary lipid levels, there was a significant increase in the activity of superoxide dismutase. The activities of total antioxidant capacity and glutathione peroxidase initially increased and then decreased significantly, peaking at prawns fed 8% and 10% lipid-level diets, respectively (*p* < 0.05). The malondialdehyde content reached its lowest point in prawns fed a diet containing 10% lipid. In addition, the mRNA expressions of hepatopancreatic diacylglycerol acyltransferase and acetyl-CoA carboxylase showed the highest values in prawns fed a 10% lipid diet. Conversely, there was a significant decrease in the mRNA expression of carnitine palmitoyltransferase-1a in the hepatopancreatic as dietary lipid levels increased. The highest mRNA expression of fatty acid-binding proteins was observed in prawns fed an 8% lipid diet. In conclusion, dietary lipid levels ranging from 8% to 11.79% are beneficial for ovarian maturation and health of *M. rosenbergii* broodstock.

## 1. Introduction

Lipids serve as the primary source of energy, providing essential fatty acids for the growth and development of aquatic animals [1]. Additionally, dietary lipids serve as precursors of hormones, eicosanoids, and enzyme cofactors for aquatic species, as well as transporters of nutrients, such as fat-soluble vitamins [2, 3]. It has also been noted that some aquatic species exhibit protein sparing effects for lipids, which can be used to decrease the amount of nitrogenous waste released into the aquatic environment from fish farm effluents and lower feed costs [4–6]. Moreover, numerous studies have demonstrated that the level of dietary lipids in aquatic animals affects their health. High-lipid diets usually lead to an increase in lipid accumulation in the body while decreasing growth performance and feed utilization [7–9]. Therefore, it is important to consider the quantitative requirements of lipids in the diet of aquatic animals.

However, it should be noted that the lipid requirements of aquatic animals vary at different growth and development stages. Previous studies have shown that lipids stored in the hepatopancreas can be translocated via the hemolymph to support ovarian development in crustaceans [10, 11]. Insufficient lipid levels will lead to oocyte atrophy and inadequate yolk accumulation in the ovary, resulting in the termination of reproductive activities [12]. Feeding diets with appropriate lipid levels during the reproductive period of aquatic animals can enhance the reproductive performance of the broodstock [13–16]. The specific lipid requirements of broodstock have been studied for various species including *Salmotrutta fario* [17], the Chinese mitten crab *Eriocheir sinensis* [73], *Penaeus stylirostris* [18], the Pacific white shrimp *Litopenaeus vannamei* [19], and others. These studies have demonstrated significant differences between the nutritional requirements of broodstock and larvae, highlighting the benefits of a diet with appropriate lipid content for gonad development, fertility, and offspring quality. Interestingly, most farms do not prioritize research and development of specialized diets for broodstock in practical production. Instead, they rely on feeding frozen baits to cultivate broodstock. However, these frozen baits are not only susceptible to bacterial contamination but also lack clear nutritional composition [20]. Therefore, it is imperative to determine the specific lipid requirement of broodstock as a basis for preparing tailored diets.

The giant river prawn *Macrobrachium rosenbergii* is an important freshwater economic prawn in our country, known for its rapid growth, strong disease resistance, and high economic value. The aquaculture industry of *M. rosenbergii* is in urgent need of rapid and high-quality development due to market demand. However, the domestic aquaculture industry of *M. rosenbergii* faces several challenges especially the lack of specialized feed. Therefore, research on the appropriate nutrient requirements for broodstock maturation will greatly promote the development of *M. rosenbergii*'s diet industry and ensure its healthy and sustainable development. The purpose of this study was to investigate the effects of dietary lipid levels on ovarian development and health of female *M. rosenbergii* broodstock and to provide a theoretical basis for the production of artificial diets for this prawn.

## 2. Materials and Methods

### 2.1. Experimental Diets

The basic diet was formulated using fish meal, soybean meal, shrimp meal, and wheat gluten meal as protein sources. Fish oil, soybean oil, and soy lecithin oil were used as lipid sources with alpha-starch serving as the carbohydrate source. This resulted in the creation of five isonitrogenous diets with varying energy levels and lipid content: 6.23% (L6%), 8.53% (L8%), 9.98% (L10%), 11.93% (L12%), and 14.48% (L14%) (Table 1). The process of formulating the experimental diets began immediately after the completion of the individual components for each diet. Initially, each ingredient was carefully mashed and sifted through a 212 µm sieve until a homogenous mass was achieved. The ingredients were then precisely weighed in accordance with the formulation using digital scales. To make dough for pelleting, all materials were thoroughly combined before adding fish oil, soybean oil, soybean lecithin oil, and distilled water. An F-26 twin-screw extruder (Institute of Chemical Engineering, South China University of Technology, Guangzhou, China) was then used to pellet this mixture. The resulting pellets with 1.5 mm were dried in a forced-air furnace at 40°C and refrigerated until they were needed.

### 2.2. Aquaculture Animals and Management

The trial lasted approximately 8 weeks, starting in September and ending in November. The breeding facility and *M. rosenbergii* broodstock were supplied by Zhejiang Zhongyi Aquatic Seed Technology Co., Ltd., located in Huzhou City, China. Prior to the trial, the cement tank and breeding equipment used for breeding were disinfected and washed. Additionally, net pieces were hung in the cement tanks to reduce the casualties caused by the attacks and competition for feed when the prawns shed their shells. *M. rosenbergii* broodstock with an initial body weight of (10.53 ± 1.97 g) were randomly divided into five groups, each consisting of four repeats, and were kept in 20 cement ponds (1.5 m³). The female-to-male ratio in the experiment was approximately 2 : 1. Every 3 days during the trial phase, one-third of the water in every tank was changed. Full feeding occurred twice a day at 8:00 a.m. and 16:30 p.m., with a feeding amount equivalent to about 1%−1.5% of body weight. The daily status of experimental prawns was recorded along with any deaths; dead prawns were promptly removed. The average water temperature varied between 23.8 and 30.8°C during the experiment, the dissolved oxygen content stayed above 6 mg/L, the pondus Hydrogenii (pH) levels varied between 7.2 and 7.6, and the ammonia nitrogen concentration remained below 0.05 mg/L. To maintain clean water conditions, food remnants and manure were cleaned up daily.

### 2.3. Sample Collection

Before sampling, the prawns were fasted for 24 h following the feeding trial. Each pond's prawns were weighed and counted under the anesthetic condition of an ice bath. Female hemolymph samples were randomly taken from each pond, and after separating serum from blood cells with a centrifuge set to 2000× *g* for 10 min at 4°C, the samples were stored at −20°C for further testing. Ovarian tissues were kept at 4% paraformaldehyde for ovarian histological analysis. Hepatopancreases tissue of females were stored at −80°C for further examination. Ovarian maturation stages of *M. rosenbergii* were observed according to the method provided by Chang and Shih [21] and Damrongphol, Eangchuan, and Poolsanguan [22].

### 2.4. Growth Performance and Ovarian Maturation Factors

The relevant indexes of growth performance and reproductive performance were calculated according to the following formulas:

  $$\begin{array}{c} \text{Survival rate }\left(\text{SR},\%\right)=100\times \text{final number of prawns}/\text{initial number of prawns}, \end{array}$$

  $$\begin{array}{c} \text{Weight gain rate}\left(\text{WGR},\%\right)=100\times \left(\text{final weight}-\text{initial weight}\right)/\text{initial weight}, \end{array}$$

  $$\begin{array}{c} \text{Specific growth rate}\left(\text{SGR},\%{\text{day}}^{-1}\right)=\left[\left(\text{Ln final body weight(g)}-\ln\text{ initial body weight(g)}\right)/\text{experimental days}\right]\times 10, \end{array}$$

  $$\begin{array}{c} \text{Gonadosomatic index}\left(\text{GSI},\%\right)=100\times \text{gonad weight}/\text{final weight}, \end{array}$$

  $$\begin{array}{c} \text{Hepatosomatic index}\left(\text{HSI},\%\right)=100\times \text{hepatosomatic weight}/\text{final weight}. \end{array}$$

### 2.5. Biochemical Parameters Analysis

The contents of serum glucose (GLU), triglyceride (TG), and total cholesterol (TC) were detected by kits from Nanjing Jiancheng Bioengineering Institute, Nanjing, China. The hepatopancreas was utilized for the analysis of biochemical enzyme activities. Total antioxidant capacity (T-AOC), superoxide dismutase (SOD) activity, glutathione peroxidase (GSH-Px), and the malondialdehyde (MDA) content were assessed using specific commercially available kits from Nanjing Jiancheng Bioengineering Institute, Nanjing, China. The levels of serum progesterone (PROG) and 17*β*-estradiol (E_2_) were detected by kits from Jiangsu Meimian Industrial Co., Ltd., China. A full-wavelength microplate reader was used to detect absorbances during the analyses (Thermo Scientific Multiskan GO 1510, Shanghai, China). As detailed in the following section, total RNA extraction was performed on the remaining hepatopancreas samples for gene analysis.

### 2.6. Study of Histology

The ovarian samples were preserved in 4% paraformaldehyde solution at 4°C for 24 h, in preparation for histological analysis. Following this, the dehydrated tissues were embedded in paraffin and sectioned into consecutive blocks of 5-μm slices. After that, these sections were viewed using Case Viewer software (3DHISTECH Ltd., Budapest, Hungary) and stained with hematoxylin and eosin (H&E). Any observed alterations within the various groups were recorded and documented accordingly.

### 2.7. Gene Expression Analysis

For gene analysis, total RNA was extracted from the hepatopancreas tissues using the Trizol technique. A protein and nucleic acid analyzer called Thermo NanoDrop 2000 was used to measure the RNA's purity and concentration. After that, the whole RNA was converted into complementary DNA (cDNA) using a reverse transcription kit (Takara, Japan) and kept for further analysis at −20°C. Through the use of quantitative real-time polymerase chain reaction (qRT-PCR), messenger RNA (mRNA) expression of genes associated with lipid metabolism in the hepatopancreas was investigated. They were acetyl-CoA carboxylase (ACC), diacylglycerol acyltransferase (DGAT), carnitine palmitoyltransferase-1 (CPT-1), and fatty acid-binding proteins (FABPs), respectively. A total of 10 μL 2 × SYBR Green Premix Ex Taq, 10 μM primers (0.2 μL each), 2 μL cDNA, and 7.6 μL H_2_O made up the 20 μL qRT-PCR reaction volume. The following were the qRT-PCR cycling conditions: 40 cycles of 10 s at 94°C, 30 s at 58°C, and 32 s at 72°C after an initial denaturation step of 10 min at 95°C. Following qRT-PCR, a melting curve was generated with an acceleration rate of 0.5°C every 5 s to validate the amplified product's specificity within the 65–95°C temperature range. *β*-Actin was chosen as the housekeeping gene in this investigation to standardize our samples because of its consistent expression. The expression levels were calculated using the 2^−*ΔΔ*CT^ method. Primers were designed using the Primer 5 software based on the sequences from National Center for Biotechnology Information (NCBI) and listed in Table 2. All primers were synthesized by Sangon Biotech Co., Ltd. (Shanghai, China).

### 2.8. Statistical Analysis

Using the Statistical Product and Service Solutions (SPSSs) 26.0 program (SPSS Inc., Chicago, IL, United States), all data analyses were carried out following the application of the Levene test to assess variance homogeneity. Next, one-way analysis of variance (ANOVA) and Tukey's test (*p* < 0.05) were used to compare the means of the experimental data. The results are presented as mean ± standard deviation (SD).

## 3. Results

### 3.1. Growth Performance

Table 3 shows the *M. rosenbergii* broodstock's growth performance. The findings indicate that the prawns were fed the 8% lipid-level diet exhibited significantly higher final weight (FW), weight gain (WG), and specific growth rate (SGR) in comparison to other groups, while the prawns fed 6% lipid-level diet showed significantly lower values than those fed other diets (*p* < 0.05; Table 3). The prawns' survival rate (SR) did not significantly differ across the dietary treatment groups (*p* > 0.05; Table 3). However, the hepatosomatic index (HSI) of female prawns increased significantly with the increase in dietary lipid level (*p* > 0.05; Table 3).

### 3.2. Serum Biochemical Indexes

There were no significant differences in serum GLU content of females among the feed treatment groups (*p* > 0.05; Table 4). But the females fed on 10% lipid-level diet exhibited significantly higher serum TG and TC content compared to all other experimental diets (*p* < 0.05; Table 4).

### 3.3. Ovarian Maturation

**Figure 1:**
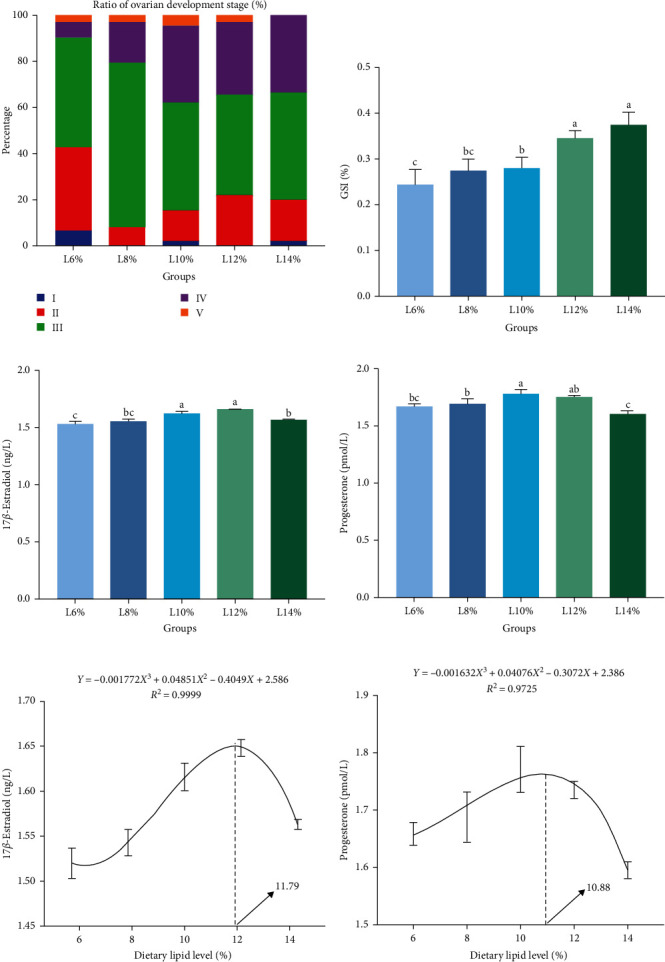
The ovarian maturation parameters of female *M. rosenbergii* broodstock fed with experimental diets for 8 weeks. (A) The proportion of prawns with ovarian development from stages I to V, (B) GSI, (C) 17*β*-estradiol (E_2_) in serum, and (D) PROG in serum. Data are mean ± SD. Different letters above bar indicate significant difference (*p* < 0.05). Polynomial regression analysis on (E) E_2_ and (F) PROG to estimate the optimal percentage of lipid level. GSI, gonadosomatic index; PROG, progesterone; SD, standard deviation.

The dietary lipid level had a significant impact on the ovarian maturation of female *M. rosenbergii* broodstock (*p* < 0.05). A large number of ovaries from the *M. rosenbergii* broodstock reach stages Ⅲ and Ⅳ after being supplemented dietary lipid levels between 8% and 14%. Throughout the experimental period, the ovaries of the prawns fed 8% and 10% lipid-level diets exhibited a better degree of development. The gonadosomatic index (GSI) of females increased when dietary lipid levels rose, and prawns fed 10%, 12%, and 14% lipid-level diets displayed significantly higher GSIs than those on 6% lipid-level diets (*p* < 0.05; Figure 1A,B). Besides, compared to the prawns fed the 6%, 8%, and 14% lipid-level diets, the PROG levels in prawns fed the 10% lipid-level diet were significantly higher (*p* < 0.05; Figure 1D). *E*_2_ content initially rose but then declined with increasing dietary lipid levels, with prawns fed the 10% and 12% lipid-level diets showing significantly higher levels than those fed other diets (*p* < 0.05; Figure 1C). The optimum lipid level required for maximum ovarian maturation was found to be 11.79% and 10.88% based on serum E_2_ and PROG content, respectively (Figure 1E,F).

### 3.4. AOC

**Figure 2:**
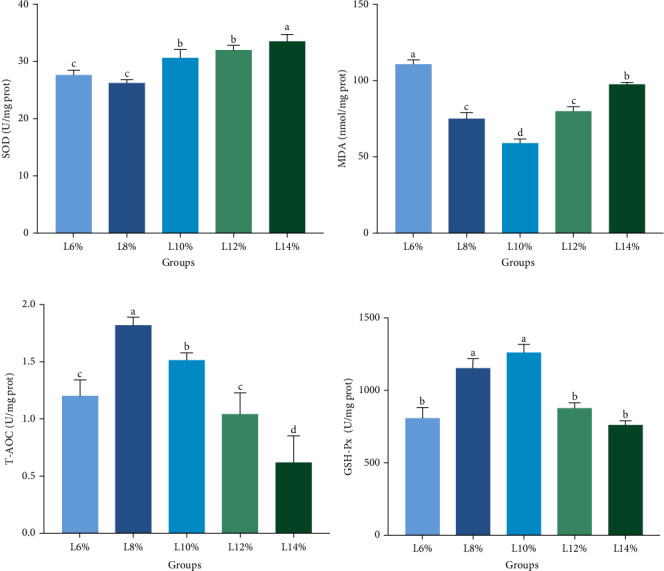
The antioxidant capacity of *M. rosenbergii* broodstock in the hepatopancreas fed with experimental diets for 8 weeks: (A) SOD, (B) MDA, (C) T-AOC, and (D) GSH-Px. Data are mean ± SD. Different letters above bar indicate significant difference (*p* < 0.05). GSH-Px, glutathione peroxidase; MDA, malondialdehyde; SD, standard deviation; SOD, superoxide dismutase; T-AOC, total antioxidant capacity.

The activity of SOD significantly increased with the rise in dietary lipid level (*p* < 0.05; Figure 2A). The MDA content in the hepatopancreas decreased significantly when the prawns were fed a 10% lipid-level diet (*p* < 0.05; Figure 2B). Moreover, the T-AOC activity of prawns fed an 8% lipid-level diet was noticeably higher than that of prawns fed other diets (*p* < 0.05; Figure 2C). The activities of GSH-Px showed a significant increase in the prawns fed 8% and 10% lipid-level diets compared to those fed other diets (*p* < 0.05; Figure 2D).

### 3.5. Histological Examination of Ovary

**Figure 3:**
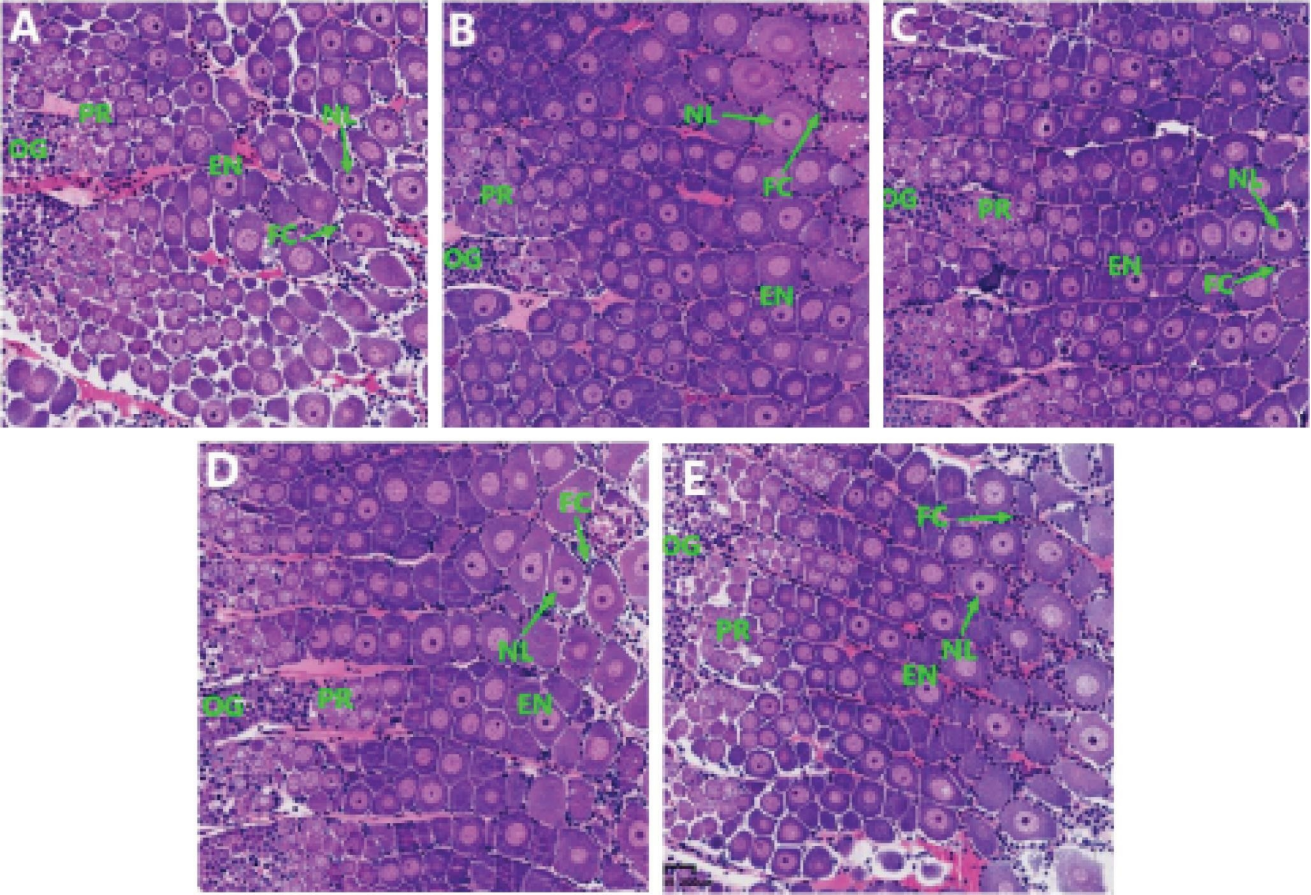
Hematoxylin and eosin staining micrographs of ovary sections of *M. rosenbergii* broodstock under different lipid levels (magnification ×100): (A) prawns fed diet including 6% lipid; (B) prawns fed diet including 8% lipid; (C) prawns fed diet including 10% lipid; (D) prawns fed diet including 12% lipid; and (E) prawns fed diet including 14% lipid. EN, endogenous vitellogenic oocytes; FC, follicular cell; NL, nucleus; OG, oogonia; PR, previtellogenic oocytes.

Figure 3 displays the H&E staining results. The ovarian histology of female *M. rosenbergii* broodstock fed 8% and 10% lipid-level diets revealed a higher presence of endogenous yolk synthesis stage oocytes (EN) with intact nucleus (NL), which were larger and exhibited a more compact arrangement. Additionally, there were more follicular cavities surrounded by follicular cells (FCs) in the 8% lipid level group compared to the other groups. Furthermore, prawns fed 10% lipid level diets displayed a greater number of previtellogenic stage oocytes (PR) when compared to the other groups.

### 3.6. Relative Expression Levels Genes

**Figure 4:**
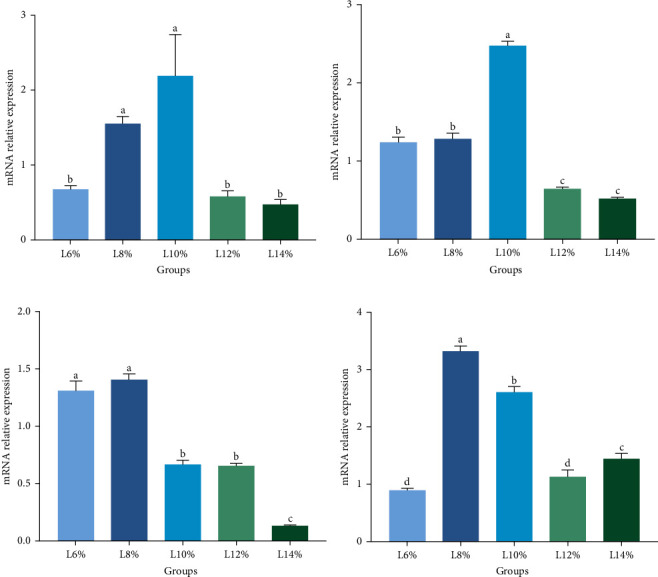
Effects of different lipid levels on the expression of (A) DGAT; (B) ACC; (C) CPT-1; and (D) FABPs in the hepatopancreas of female *M. rosenbergii* broodstock. Data are mean ± SD. Different letters above bar indicate significant difference (*p* < 0.05). ACC, acetyl-CoA carboxylase; CPT-1, carnitine palmotoyltransferase-1; DGAT, diacylglycerol acyltransferase; FABPs, fatty acid-binding proteins.

Figure 4 illustrates the expression of genes related to lipid metabolism. In comparison to the prawns fed 6%, 12%, and 14% lipid-level diets, the expression of the gene DGAT, which is connected to triacylglycerol biosynthesis, was significantly higher in prawns fed 8% and 10% lipid-level diets (*p* < 0.05; Figure 4A). Additionally, the expression of fatty acid synthesis-related gene ACC initially increased and then decreased in response to rising lipid levels, peaking in the prawns fed a 10% lipid-level diet (Figure 4B). Furthermore, CPT-1 expression decreased markedly as lipid levels increased, with the prawns fed 6% and 8% lipid-level diets exhibiting much higher CPT-1 expression than those fed other diets (*p* < 0.05; Figure 4C). Moreover, the intracellular fatty acid transport-related gene FABP was significantly upregulated in response to an increase in dietary lipid levels, particularly evident in the prawns fed an 8% lipid-level diet (*p* < 0.05; Figure 4D).

## 4. Discussion

The present study demonstrated that the growth performance of *M. rosenbergii* broodstock was significantly higher when fed diets containing 8%–14% lipid levels compared to the 6% lipid-level diet, of which the prawns fed 8% lipid-level diet showed the best growth performance. This suggests that an appropriate dietary lipid level promotes the growth of *M. rosenbergii* broodstock. Similar positive effects of an appropriate dietary lipid level on the growth performance were also observed in *Procambarus clarkia* broodstock and yellow catfish *Pelteobagrus fulvidraco* broodstock [23, 24]. Additionally, the HSI of female prawns increased significantly with an increase in dietary lipid levels, as increased dietary lipid levels can promote an increase in the amount of lipids in the hepatopancreas and enlargement of fat cells [25–27]. Therefore, lipids not only serve as the main energy source for promoting growth but are also closely related to lipid metabolism in the hepatopancreas of broodstock. As female broodstock develops, their ovaries also require a large accumulation of lipids to promote maturation [28–30]. The GSI is frequently used as an essential indicator to evaluate gonad development in aquatic animals [31]. The present study also showed that the GSI in prawns fed 10%–14% lipid level diets was higher than those containing 6%, indicating the GSI increased with the higher dietary lipid levels, similar to results in snakehead murrel *Channa striatus* [32]. During gonadal development, nutrients from the hepatopancreas are transferred to the ovaries, resulting in a decrease in HSI and an increase in GSI [11]. However, there was no negative correlation between GSI and HSI observed in this experiment, which is consistent with the results obtained for *L. vannamei* and may be due to nonsynchronous accumulation and transport of nutrients within the hepatopancreas and ovaries [33].

Previous studies have demonstrated that the dietary lipid level can affect the secretion of reproductive hormones in the body [12, 34]. Additionally, an appropriate lipid level has been found to effectively increase the production of steroid hormones crucial for ovarian development [12]. Consistent with previous findings, our study found that the serum *E*_2_ hormone levels in the prawns fed 10% and 12% lipid level diets were significantly higher than those in other diets groups, and the prawns fed with a lipid level between 10% and 12% also exhibited higher serum PROG levels compared to those fed a 14% lipid level diet. Estradiol and PROG as major sex steroid hormones play a crucial role in regulating reproductive endocrine processes and can significantly affect ovarian development and reproductive behavior in female prawns [35]. During ovarian development, E_2_ and PROG could stimulate ovarian maturation and yolk protein synthesis in the ovary of *L. vannamei* [36, 37]. Thus, it is inferred that an appropriate dietary lipid level could promote the secretion of sex hormones in *M. rosenbergii* broodstock, thereby promoting its gonadal development. Moreover, our study found that dietary lipid levels not only affect the secretion of serum steroid hormones but also impact ovarian morphology in female *M. rosenbergii* broodstock. The ovary consists of five developmental stages of oocytes: oogonia (OG), previtellogenic oocytes (PR), endogenous vitellogenic oocytes (EN), exogenous vitellogenic oocytes (EX), and mature oocytes (MO) [38, 39]. According to this classification system, we observed that a higher number of ovaries from the *M. rosenbergii* broodstock reaching stage Ⅲ (endogenous vitellosynthesis oocytes) and stage Ⅳ (exogenous vitellosynthesis oocytes) after being supplemented with dietary lipid levels between 8% and 14%. This suggests that dietary lipid levels could have an impact on ovarian maturation. Prior studies have also revealed that dietary lipid levels can influence ovarian maturation or morphology in female broodstock [40, 41]. In our study, it is evident that there were more EN in prawns fed 8% and 10% lipid-level diets, with a more compact arrangement compared to the less tightly arranged structure of the ovarian tissue in prawns fed other lipid-level diets. This outcome further demonstrates that a suitable level of lipid in the diet could advance oocyte growth and development [42], and maintain good histological morphology of the ovary, thus effectively contributing to its development and maturation in *M. rosenbergii* broodstock.

During the reinforcement period of crustacean broodstock, lipids from the hepatopancreas can be transferred via the hemolymph to assist in the development of the ovaries in crustaceans [10, 11]. Lipids play a crucial role as the primary nutrient for maintaining the integrity of ovarian tissue membranes and serve as an essential energy source for ovary development during oocyte maturation through lipid metabolism [43]. Lipid metabolism in organisms is primarily influenced by lipid synthesis, transport, and catabolism [44]. ACC serves as the rate-limiting enzyme that catalyzes the first phase of fatty acids anabolism, resulting in the synthesis of malonyl monoacyl coenzyme A from acetyl coenzyme A [45]. According to the results of the current study, there is a considerable upregulation of ACC expression when dietary lipid levels are between 6% and 10%, and a downregulation when dietary lipid levels are higher than 10%. Similar results have been observed in juvenile greasyback shrimp *Metapenaeus ensis* [46], golden pompano *Trachinotus ovatus* [47], and grass carp *Ctenopharyngodon idellus* [2, 3]. This suggests that proper dietary lipids can promote lipid synthesis of *M. rosenbergii* broodstock. Furthermore, the expression of DGAT, involved in the triacylglycerol biosynthesis [48], also showed the highest level in females fed 10% lipid. These results are consistent with the highest content of TG and TC in the serum of prawns fed 10% lipid-level diets in our study. The findings underscore the intricate feedback mechanisms that govern the homeostasis of lipids in crustaceans [49, 50]. Within a specific range of dietary lipid absorption, there is a gradual upregulation of lipogenic gene expression to uphold optimal lipid levels. Once this threshold is surpassed, there is a downregulation of lipogenic gene expression, thereby attenuating the pace of lipogenesis and preventing excessive lipid accumulation within the body [51]. The transport and absorption of fatty acids are largely facilitated by the protein known as FABPs, which exhibit high affinity for fatty acids [44, 46, 52]. Notably, when dietary lipid levels rose above 10%, FABP expression was downregulated in this study. This phenomenon is consistent with a study on juvenile mud crab *Scylla paramamosain* [53], suggesting that excessive dietary lipid intake inhibits fatty acid absorption by hepatopancreatic cells. Fatty acid *β*-oxidation occurs in mitochondria via carnitine, with CPT-1 being a key enzyme in this process [54, 55]. The present study observed a down-regulation of CPT-1 expression when dietary lipid levels rose from 8% to 14%. This suggests that decreased transfer of acyl-carnitine across the outer membrane of the mitochondria prevents long-chain fatty acids from entering the mitochondria's matrix, which in turn reduces *β*-oxidation. Previous research has shown that diminished CPT-1 activity primarily contributes to reducing the ability of hepatic *β*-oxidation [56]. To promote quick ovarian growth, the developing ovaries receive an adequate amount of long-chain fatty acids, which lowers the *β*-oxidation process of these fatty acids at this time [57]. Therefore, inhibited *β*-oxidation in the female prawn's hepatopancreas may be indicative of an adaptive mechanism aimed at conserving fatty acids for enhanced ovarian growth. Interestingly, there was no variation in serum GLU content among *M. rosenbergii* broodstock based on dietary lipid levels, suggesting that dietary lipid rather than carbohydrate serves as the primary source of metabolic organic energy for egg development during ovarian development [58].

Oxidative responses can be impacted by dietary lipid imbalances [59]. Furthermore, long-term high-lipid diets have a risk of causing an overabundance of reactive oxygen species (ROS) generation and increased cellular lipid deposits [60], which can damage cell membranes and ultimately cause permanent alterations in cells and tissues [61]. Lipid peroxidation may occur concurrently with the change in lipid deposition, as reflected by the content of MDA, which serves as an indicator of free radical damage [62]. SOD can convert superoxide radicals to peroxide, while GSH-Px is capable of detoxifying peroxide and organic hydroperoxide [63–65], with T-AOC serving as an important marker of AOC [66]. In this study, hepatopancreas MDA contents reached the lowest in prawns fed on 10% lipid level diet. Additionally, in the hepatopancreas, T-AOC and GSH-Px generally showed an initial increase followed by a decrease with increased lipid levels. Similar findings were noted for *L. vannamei*, where the activity of antioxidant enzymes was reduced by low or high lipid levels [67]. However, the SOD activity increased with the increase of dietary lipid level, reaching its peak in the prawns fed 14% lipid level diet. This finding is not surprising, considering the essential role of SOD as an endogenous antioxidant. By enhancing its activity, it can effectively counteract the body's increased generation of ROS, thereby providing protection against oxidative stress-induced damage [68]. A similar situation was observed among other crustaceans like *S. paramamosain* and juvenile oriental river prawn *Macrobrachium nipponense* [53, 69, 70], where appropriate dietary lipid intake enhanced coping abilities against oxidative stress by raising SOD activities while lowering MDA contents [71]. To sum up, prawns consuming high levels of lipids experience two effects: nutritional intake and heightened susceptibility to peroxidation, potentially compromising their overall well-being [72].

## 5. Conclusion

The findings of the present study suggest that a dietary lipid level ranging from 8% to 11.79% is adequate to meet the nutritional requirement of female *M. rosenbergii* broodstock during the reinforcement stage of ovarian development. This range has the potential to improve growth performance, enhance AOC, optimize lipid metabolism, and influence the synthesis of serum steroid hormones in female *M. rosenbergii* broodstock, thereby promoting ovarian maturation without compromising overall health.

## Figures and Tables

**Table 1:** Ingredient formulation (g/kg dry basis) and proximate composition (% dry weight) of the experimental diets fed to *M. rosenbergii* broodstock.

Ingredients	Experimental diets				
	L6%	L8%	L10%	L12%	L14%
Fish meal	35	35	35	35	35
Soybean meal	10	10	10	10	10
Shrimp meal	10	10	10	10	10
Wheat gluten meal	15	15	15	15	15
Fish oil	0.2	1.2	2.2	3.2	4.2
Soybean oil	0.2	1.2	2.2	3.2	4.2
Soy lecithin	2	2	2	2	2
Alpha-starch	10	10	10	10	10
Cholesterol	0.5	0.5	0.5	0.5	0.5
Vitamin premix^a^	0.5	0.5	0.5	0.5	0.5
Mineral premix^b^	0.5	0.5	0.5	0.5	0.5
Choline chloride	0.5	0.5	0.5	0.5	0.5
Calcium dihydrogen phosphate	1.5	1.5	1.5	1.5	1.5
Sodium carboxymethyl cellulose	2	2	2	2	2
Cellulose	12.1	10.1	8.1	6.1	4.1
Total	100	100	100	100	100
Proximate composition					
Dry matter (%)	92.86	92.52	92.41	92.33	92.68
Crude protein (%)	46.93	46.88	46.86	46.93	46.72
Crude oil (%)	6.23	8.53	9.98	11.93	14.48
Ash content (%)	7.46	7.69	7.53	7.58	7.82

^a^Vitamin premix contained the following (per kg): VA 6.4 g, VC 151.52 g, VE 71 g, VD_3_ 2 g, VB_1_ 3.2 g, VB_2_ 9 g, VB_6_ 4 g, VB_12_ 0.08 g, nicotinic acid 16 g, folic acid 1 g, inositol 64 g, biotin 0.2 g, calcium pantothenate 14 g, and zeolite powder 777.02 g.

^b^Mineral premix contained the following (per kg): CuSO_4_ · 5H_2_O 1.95 g, ZnSO_4_ · 7H_2_O 30.91 g, MnSO_4_ · H_2_O 5.23 g, FeSO_4_ · 7H_2_O 24.83 g, Ca(IO_3_)_2_ 0.46 g, Na_2_SeO_3_ 0.09 g, CoCl_2_ · 6H_2_O 0.32 g, and MgSO_4_ · 7H_2_O 244.9 g.

**Table 2:** Nucleotide sequences for real-time PCR primers.

Gene	GeneBank accession number	Primer sequences (5′−3′)	Product length (bp)
DGAT	XM_067108401.1	GCCTTTTGCTGAAATGGCTGAG AGGTGTCCCTGTAGGCGAAGA	164

ACC	XM_067090789.1	GATGAGGGATTCAAGCCCAGTT TCCCTGTCTTCACCCCACGA	158

FABP	XM_067082281.1	AACGACGAATGGACGCTGAA TTCCCTTAGTGGCGTTCTGG	169

CPT-1	XM_067095934.1	AGATTGCCTCTGCCTGC AAAGCAGCGTGGGTGAC	164

*β*-Actin	AY651918.2	TCCGTAAGGACCTGTATGCC TCGGGAGGTGCGATGATTTT	136

Abbreviations: ACC, acetyl-CoA carboxylase; CPT-1, carnitine palmotoyltransferase-1; DGAT, diacylglycerol acyltransferase; FABPs, fatty acid-binding proteins.

**Table 3:** Growth performance of *M. rosenbergii* broodstock fed different dietary lipid levels.

Treatment	Diets (lipid level %)				
	L6%	L8%	L10%	L12%	L14%
FW (g)	19.43 ± 0.18^c^	22.98 ± 0.09^a^	21.60 ± 0.19^b^	21.14 ± 0.59^b^	22.06 ± 0.60^ab^
WG (%)	103.64 ± 5.29^c^	134.08 ± 5.35^a^	121.07 ± 6.42^b^	120.66 ± 1.54^b^	126.98 ± 10.00^ab^
SGR (%)	1.05 ± 0.04^c^	1.66 ± 0.09^a^	1.21 ± 0.17^b^	1.23 ± 0.10^b^	1.39 ± 0.17^ab^
SR (%)	65.55 ± 3.85	67.78 ± 5.09	72.22 ± 1.92	72.25 ± 5.04	71.11 ± 5.09
HSI (%)	6.46 ± 0.51^c^	7.15 ± 0.66^b^	7.40 ± 0.41^b^	7.56 ± 0.37^ab^	8.14 ± 0.52^a^

*Note:* Values are mean ± SD (*n* = 3). Values in the same column with different superscripts letters indicate significant difference (*p*  < 0.05).

Abbreviations: FW, final weight; HSI, hepatosomatic index; SD, standard deviation; SGR, specific growth rate; SR, survival rate; WG, weight gain.

**Table 4:** Serum biochemical indexes of female *M. rosenbergii* broodstock fed different dietary lipid levels.

Treatment	Diets (lipid level %)				
	L6%	L8%	L10%	L12%	L14%
GLU (mmol/L)	1.88 ± 0.05	1.96 ± 0.10	1.57 ± 0.03	1.56 ± 0.51	1.66 ± 0.03
TG (mmol/L)	3.32 ± 0.05^d^	5.27 ± 0.09^b^	6.00 ± 0.08^a^	3.46 ± 0.07^d^	4.45 ± 0.03^c^
TC (mmol/L)	6.06 ± 0.07^c^	6.86 ± 0.18^b^	7.57 ± 0.21^a^	5.63 ± 0.20^cd^	5.91 ± 0.25^cd^

*Note:* Values are mean ± SD (*n* = 3). Values in the same column with different superscripts letters indicate significant difference (*p*  < 0.05).

Abbreviations: GLU, glucose; SD, standard deviation; TC, total cholestrol; TG, triglyceride.

## Data Availability

The data that support the findings of this study are available upon request from the corresponding author. The data are not publicly available due to privacy or ethical restrictions.
